# The Role of Bariatric Arterial Embolization in Obesity Management: A Narrative Review

**DOI:** 10.7759/cureus.97157

**Published:** 2025-11-18

**Authors:** John Salib, Mark Salib, Shanu Sivakumar, Aryan Kahlon, Matthew Phillips

**Affiliations:** 1 School of Medicine, St. George's University School of Medicine, St. George's, GRD; 2 General Surgery, Community First Medical Center, Chicago, USA

**Keywords:** appetite regulation, bariatric arterial embolization (bae), endovascular treatment, ghrelin suppression, interventional radiology, minimally invasive therapy, obesity management, weight loss intervention

## Abstract

Obesity remains one of the most pressing global health challenges, contributing to significant metabolic, cardiovascular, and psychosocial burdens. Traditional interventions such as diet modification, pharmacologic therapy, and bariatric surgery often face limitations in effectiveness, accessibility, and patient acceptance. This qualitative narrative review examines bariatric arterial embolization (BAE), a minimally invasive endovascular technique that targets the gastric fundus to suppress ghrelin secretion and promote appetite regulation. By reducing hormonal drive to eat, BAE aims to achieve weight loss through physiological modulation rather than anatomical alteration. Evidence from clinical studies demonstrates that BAE produces meaningful short-term weight reduction. The procedure is generally well-tolerated, with transient abdominal discomfort and nausea being the most frequent minor side effects, and serious complications remaining uncommon. Compared to surgical approaches, BAE offers lower procedural risk, faster recovery, and broader applicability for patients seeking non-surgical solutions; however, its long-term durability and metabolic sustainability require further investigation. Overall, this review highlights BAE as a promising addition to the continuum of obesity management, bridging the gap between conservative and surgical interventions. Its minimally invasive, hormonally driven mechanism provides a feasible pathway for weight control and metabolic improvement, underscoring the need for continued research into standardized techniques, patient selection criteria, and long-term outcomes.

## Introduction and background

Obesity is a primary global health concern, with prevalence rates having more than tripled since 1975 and current estimates indicating that over 650 million adults are affected worldwide [1]. It is increasingly recognized as a complex, multifactorial disease marked by excessive adiposity and impaired regulation of energy balance. As a result, obesity significantly elevates the risk of cardiovascular disease, type 2 diabetes mellitus, nonalcoholic fatty liver disease, obstructive sleep apnea, and several malignancies [2]. Beyond its impact on individual health, the growing burden of obesity reduces life expectancy, diminishes quality of life, and places substantial economic pressure on healthcare systems [3].

Conventional management strategies, including dietary modification, increased physical activity, and behavioral interventions, often yield only modest and temporary weight loss, with relapse remaining common [4]. Pharmacologic therapy can benefit certain patients but is limited by variable efficacy, adverse effects, and concerns about long-term safety [5]. Bariatric surgery continues to offer the most reliable and durable weight reduction, along with improvements in obesity-related comorbidities [6]. Despite its proven efficacy, bariatric surgery remains underused due to operative risk, high costs, limited access, and patient reluctance towards invasive procedures. These issues have identified a need for an effective, less invasive intervention that can bridge the gap between conservative measures and surgery.

Bariatric arterial embolization (BAE) has emerged as a promising endovascular technique designed to influence appetite and energy regulation by targeting the hormonal pathways involved in obesity. The procedure selectively embolizes branches of the left gastric artery supplying the gastric fundus, the primary source of circulating ghrelin [7]. By reducing blood flow in this region, BAE lowers ghrelin production, suppresses appetite, and facilitates weight loss. Initial preclinical and clinical studies have reported encouraging results regarding safety, feasibility, and short-term efficacy [8-10]. Nevertheless, the durability of these outcomes and the long-term safety profile remain uncertain.

This narrative review aims to summarize the current evidence surrounding BAE, with emphasis on its physiological rationale, technical aspects, reported outcomes, and potential role in the future management of obesity.

## Review

Methods

**Figure 1:**
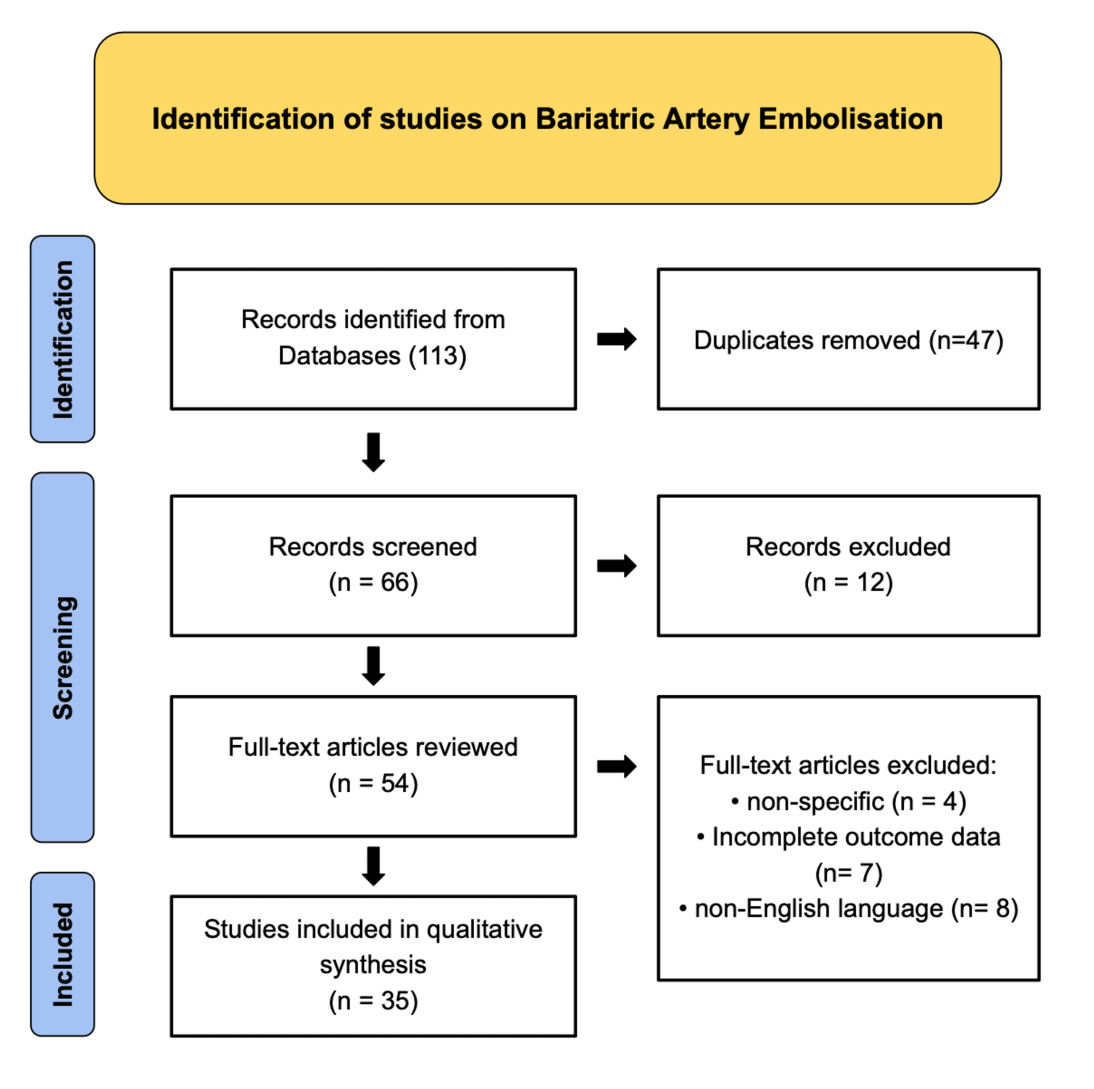
PRISMA Flow Diagram of Study Selection for Bariatric Artery Embolization. Flowchart illustrating the systematic identification, screening, eligibility, and inclusion process for studies evaluating Bariatric Artery Embolisation. A total of 113 records were identified through database searching, with 47 duplicates removed. After screening 66 records, 12 were excluded based on titles and abstracts. Of the 54 full-text articles reviewed, 19 were excluded for reasons including non-specific focus (n = 4), incomplete outcome data (n = 7), and non-English language (n = 8). Ultimately, 35 studies were included in the qualitative synthesis. PRISMA: Preferred Reporting Items for Systematic Reviews and Meta-Analyses

This review followed a narrative approach while incorporating Preferred Reporting Items for Systematic Reviews and Meta-Analyses (PRISMA)-based methods to enhance clarity, transparency, and reproducibility (as depicted in Figure 1). A structured literature search was conducted across PubMed, MEDLINE, and Google Scholar to identify relevant English-language studies published between January 2000 and October 2025. The search strategy combined Medical Subject Headings (MeSH) and free-text terms related to bariatric embolization, including “bariatric arterial embolization,” “left gastric artery embolization,” “ghrelin suppression,” “weight loss intervention,” “endovascular obesity therapy,” “gastric fundal embolization,” “non-surgical obesity management,” “ghrelin modulation,” “angiographic techniques,” and “metabolic outcomes.” Boolean operators (AND, OR, NOT) were applied to refine precision and ensure comprehensive retrieval of relevant sources.

Five blinded reviewers independently screened all studies to minimize selection and interpretation bias. Each reviewer assessed titles, abstracts, and full texts using predefined criteria for eligibility. Extracted data included study design, population characteristics, embolization technique, target vessel, embolic material, metabolic and hormonal outcomes, complications, and follow-up duration (as depicted in Table 1). Disagreements were resolved through discussion and consensus to ensure consistency.

**Table 1:** Summary of Clinical and Preclinical Studies on Bariatric Arterial Embolization for Obesity Management. This table summarizes published literature on bariatric arterial embolization and left gastric artery embolization, including study design, patient population, procedural context, and main findings. Both human and animal studies are included, highlighting weight loss outcomes, hormonal modulation (primarily ghrelin suppression), procedural feasibility, and follow-up duration. The table includes systematic reviews, pilot studies, prospective trials, narrative reviews, and preclinical studies to provide a comprehensive overview of the current evidence base for bariatric arterial embolization and left gastric artery embolization as minimally invasive interventions for obesity [1-35].

Author (Year)	Study Design/Population	Procedure/Context	Main Findings
Ravetta P, Kebbou T, Poras M (2023)	Narrative review	Bariatric artery embolization (BAE) in obese patients	Summarizes clinical and preclinical data, including procedural techniques, patient selection, weight loss outcomes, and hormonal changes (ghrelin suppression) observed in studies [1].
Kordzadeh A, Lorenzi B, Hanif MA, et al. (2018)	Systematic review	Left gastric artery embolization (LGAE)	Reported short-term weight loss of 5–10% body weight at 1–12 months follow-up; procedure shown to reduce ghrelin levels; overall minimally invasive and technically feasible [2].
Anton K, Rahman T, Bhanushali A, Patel AA (2016)	Review	Bariatric LGAE	Highlighted gut hormone modulation (ghrelin, leptin) as the key mechanism; preclinical and early clinical trials showed modest weight loss and reduced appetite [3].
Weiss CR, Bailey CR (2020)	Editorial / commentary	BAE readiness	Early human trials show feasibility and short-term weight loss; emphasizes lack of long-term data and need for randomized trials [4].
Patel P, Thomas R, Hamady M, et al. (2023)	Study protocol (EMBIO trial)	LGAE in BMI 35–50	Describes trial design for standardized assessment of efficacy, safety, hormonal effects, and imaging endpoints; aims to provide robust human evidence [5].
Weiss CR, Kathait AS (2017)	Review	BAE in obese patients	Summarized clinical trials showing 5–12% weight loss at 6–12 months; hormonal changes correlated with appetite suppression [6].
Aldawudi I, Katwal PC, Jirjees S, et al. (2020)	Review	Clinical trials of BAE	Included all recent clinical trials; weight loss ranged from 4 to 11% at 6–12 months; ghrelin reduction consistent; highlighted need for standardized protocols [7].
Pirlet C, Ruzsa Z, Nemes B, et al. (2020)	Observational	LGAE	Sustained weight loss at 1–2 years (~8–12% total body weight); procedure technically successful in all patients; long-term hormonal modulation observed [8].
Zhong B, Abiola G, Weiss CR (2018)	Review	BAE	Early human trials demonstrated 5–10% weight reduction at 3–12 months; low procedural risk; identified need for larger trials to confirm durability [9].
Fu Y, Kraitchman DL (2020)	Preclinical review	BAE animal models	Animal studies showed ghrelin suppression, reduced food intake, and slowed gastric emptying; provided mechanistic rationale for human translation [10].
Hafezi-Nejad N, Bailey CR, Weiss CR (2020)	Narrative review	Human trials of BAE	Summarized clinical studies reporting 5–12% weight loss, decreased ghrelin levels, and improved metabolic parameters over 6–12 months [11].
Shoar S, Saber AA, Aladdin M, et al. (2016)	Systematic review	Gastric artery manipulation	Evaluated preclinical and clinical data; LGA embolization consistently led to modest weight loss; appetite reduction linked to hormonal changes [12].
Gilchrist IC (2019)	Commentary	Catheter-based BAE	Conceptual discussion of potential catheter-based techniques; emphasizes the need for clinical validation and safety assessment [13].
Khurana R, Pandey NN, Kumar S, et al. (2023)	Systematic review & meta-analysis	BMI 25–40	Pooled analysis: 4–10% weight loss over 3–12 months; improvement in metabolic parameters in overweight and mildly obese patients; procedure technically feasible [14].
Wang Z, Cao QY, Xiang C, et al. (2024)	Animal study (dogs)	BAE	Slowed gastric emptying, improved postprandial glycemia, and reduced food intake in obese dogs with impaired glucose tolerance; suggests metabolic benefit beyond weight loss [15].
Syed M, Efridi W, Khan T, et al. (2021)	Case report	Catheterization technique for LGAE	Described a novel approach for selective left gastric artery catheterization; enabled accurate microsphere delivery; improved procedural efficiency [16].
Reddy VY, Neužil P, Musikantow D, et al. (2020)	Multicenter trial	Transcatheter BAE	Demonstrated 6–12% weight reduction at 6–12 months; procedure technically successful in all cases; ghrelin reduction observed in early follow-up [17].
Mizandari M, Keshavarz P, Azrumelashvili T, et al. (2021)	Systematic review & meta-analysis	Human & animal studies	Weight loss outcomes 4–12% over 3–12 months; ghrelin levels consistently decreased; procedure feasible in animal and human studies [18].
Okida LF, Henrique J, Sarmiento-Cobos M, et al. (2020)	Observational	Bariatric surgery post-cardiac revascularization	Bariatric surgery was safe in patients with prior cardiac interventions; outcomes similar to the general population; supports surgical feasibility [19].
Elsaid MI, Li Y, Bridges JFP, et al. (2022)	Observational	Bariatric surgery in severe obesity + NAFLD	Bariatric surgery improved cardiovascular outcomes, reduced hospitalizations, and lowered the risk of metabolic complications [20].
Marrone AK (2020)	Review	FDA perspective on weight-loss devices	Discusses device approval, safety monitoring, and clinical trial requirements for IR-guided obesity treatments [21].
Esparham A, Shoar S, Mehri A, et al. (2023)	Propensity-matched analysis	Bariatric surgery in pulmonary hypertension	Surgery associated with improved cardiovascular outcomes; demonstrates feasibility in high-risk patients [22].
di Giuseppe R, Hansel B, Puyraimond Zemmour J, et al. (2024)	Observational	LGAE	Reported 6–12% weight loss at 6–12 months; technical success achieved in all patients; confirms short-term effectiveness [23].
Gunn AJ, Weiss CR (2019)	Commentary	Diabetic patients	Discussed potential for BAE to improve glycemic control via ghrelin modulation; suggested the need for targeted diabetic trials [24].
Elens S, Roger T, Elens M, et al. (2018)	Prospective pilot	Gastric embolization	Demonstrated weight loss of 5–8% over 6 months in overweight patients; procedure safe and feasible [25].
Latif MA, Tunacao JM, Fu Y, et al. (2023)	Trial analysis (BEAT Obesity)	BAE	Hormonal analysis showed correlation between ghrelin suppression and weight loss; supports the mechanistic role of appetite regulation [26].
Zhong BY (2019)	Commentary	BAE	Proposed ideal patient characteristics: overweight or moderately obese without significant comorbidities; emphasizes personalized selection [27].
Weiss CR, Abiola GO, Fischman AM, et al. (2019)	BEAT Obesity trial	Arterial embolization	1-year follow-up: 7–10% weight loss; sustained ghrelin reduction; procedural feasibility confirmed [28].
Levigard RB, Serrão H, de C, et al. (2021)	Pilot study	BMI 30–39.9 + metabolic syndrome	Weight reduction of 6–9% at six months; improved metabolic parameters (lipids, glucose); technically successful in all patients [29].
Fu Y, Abiola G, Tunacao J, et al. (2023)	Preclinical	Embolic microsphere sizing	A preclinical study showed that the optimal microsphere size improved distal delivery and procedural efficacy in animal models [30].
Kipshidze N, Archvadze A, Bertog S, et al. (2015)	Review	Endovascular bariatrics	Conceptual review; discussed procedural techniques, potential efficacy, and translational challenges [31].
Bai Z, Qin Y, Deng G, et al. (2017)	Pilot study	LGAE	5 patients followed for 9 months; achieved 5–8% weight loss; ghrelin reduction observed [32].
Pirlet C, Ruzsa Z, Costerousse O, et al. (2018)	Pilot study	Transradial LGAE	Demonstrated procedural feasibility and short-term weight loss of 5–10%; minimally invasive access successful [33].
Jernigan SR, Osborne JA, Buckner GD (2020)	Benchtop study	Catheter type & injection method	Showed how catheter selection and injection technique influence microsphere distribution; informs procedural optimization [34].
Diana M, Pop R, Beaujeux R, et al. (2015)	Animal study (porcine)	EMBARGO technique	Proof-of-concept study; selective LGA embolization feasible in the porcine model; showed ghrelin reduction and decreased food intake [35].

Studies were included if they involved human or animal subjects and investigated the feasibility, safety, physiological mechanisms, or metabolic outcomes of BAE or left gastric artery embolization (LGAE). Eligible study designs comprised prospective and retrospective clinical trials, cohort studies, case series, systematic or narrative reviews, and preclinical experimental models presenting quantifiable findings. Exclusion criteria encompassed non-English publications, conference abstracts lacking complete data, editorials, commentaries, duplicate records, and studies without measurable endpoints such as weight change, hormonal modulation, or metabolic parameters. This selection strategy prioritized methodological rigor while preserving the breadth of evidence across translational and clinical domains.

All extracted data were synthesized qualitatively to provide a balanced overview of current evidence, highlight consistent findings, and identify gaps requiring further investigation. The inclusion of multiple independent reviewers, predefined selection criteria, and consensus-based resolution strengthened the methodological integrity and ensured a transparent, reproducible synthesis of the literature.

Physiology of ghrelin and appetite regulation

Ghrelin is a 28-amino acid peptide hormone predominantly secreted by the stomach, with its biologically active acylated form generated through the action of ghrelin O-acyltransferase (GOAT) [11,12]. It is the only known circulating orexigenic hormone and plays a central role in appetite regulation and energy homeostasis. Circulating levels rise during fasting and peak preprandially, serving as a hunger signal to the brain, and then fall rapidly after food intake [13].

In the hypothalamic arcuate nucleus, ghrelin acts via the growth hormone secretagogue receptor type 1a (GHSR1a) expressed on agouti-related peptide (AgRP) and neuropeptide Y (NPY) neurons. Activation of these pathways stimulates feeding behavior, promotes adiposity, and counteracts the anorexigenic actions of leptin, primarily through downstream NPY/Y1 receptor signaling [12,14,15].

The effects of ghrelin on food intake are immediate and biphasic. Both endogenous secretion and exogenous administration elicit acute increases in feeding as well as longer-term enhancement of adiposity [11-13]. Regulation of ghrelin release is mediated by vagal and sympathetic neural inputs in addition to metabolic cues. Suppression occurs after meals, primarily through the actions of insulin and nutrient-derived signals from the small intestine, rather than direct gastric nutrient contact [11-14].

**Figure 2:**
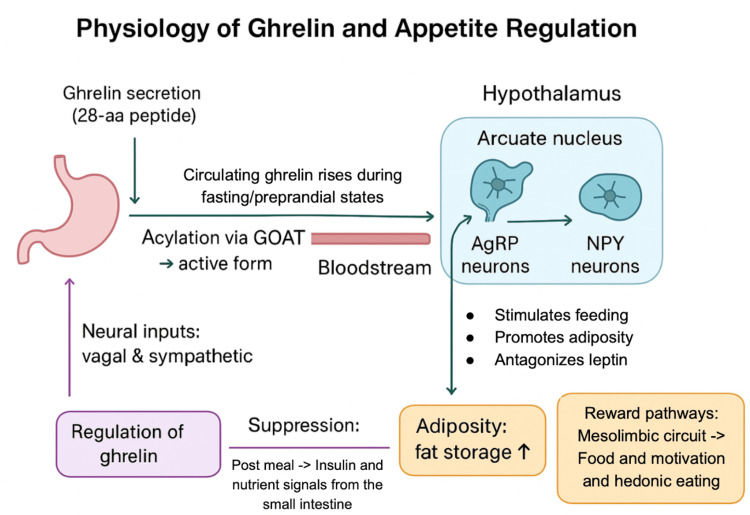
Physiology of Ghrelin and Its Role in Appetite Regulation. Ghrelin, secreted mainly by the stomach and activated via GOAT, rises during fasting and signals hunger through AgRP and NPY neurons in the hypothalamic arcuate nucleus. This promotes feeding, adiposity, and reward-driven eating while antagonizing leptin. Ghrelin release is regulated by neural and metabolic cues and suppressed after meals. These pathways highlight the hormone’s central role in energy homeostasis and its relevance as a therapeutic target in obesity management [11-14]. Figure created and designed by the authors. GOAT: Ghrelin O-acyltransferase; AgRP: agouti-related peptide; NPY: neuropeptide Y

Beyond its orexigenic properties, ghrelin contributes to energy balance through multiple mechanisms. It enhances fat storage, modulates glucose metabolism, and influences mesolimbic reward pathways that drive food motivation and hedonic eating. Through these central and peripheral actions (as depicted in Figure 2), ghrelin functions as an integrative signal linking gastrointestinal activity with central nervous system circuits to maintain overall energy homeostasis [11-14].

Concept of bariatric arterial embolization

The first reported use of LGAE was in 1973 for the treatment of gastric bleeding, rather than weight loss [16]. Its metabolic potential was recognized decades later when retrospective analyses of patients undergoing embolization for gastrointestinal bleeding revealed incidentally unintended weight reduction [16]. This unforeseen clinical outcome provided novel insight into the significance of fundal perfusion and its influence on body weight. Animal studies subsequently demonstrated that BAE lowers circulating ghrelin, reducing appetite and body weight [17]. Early human trials, though limited in size, have shown strong promise towards the feasibility of the procedure and modest weight loss outcomes [18].

Conceptually, BAE is considered a metabolic intervention, sharing with bariatric surgery the goal of suppressing ghrelin to decrease appetite and support weight loss. It is being explored as an intermediate option between pharmacotherapy and established operations such as sleeve gastrectomy (SG), Roux-en-Y gastric bypass (RYGB), and adjustable gastric banding [19]. Although bariatric procedures demonstrate strong efficacy, they remain associated with procedural risks, such as anastomotic leaks occurring in approximately 0.1% to 5.6% of cases, intussusception in about 1%, gallstone formation in 13% to 36%, and, in some instances, the need for reoperation [18,19]. Furthermore, patients who have enacted lifestyle modifications, however, do not qualify for bariatric procedures and are currently restricted to only a few medications, which offer only modest weight loss [20]. In contrast, BAE offers a long-term, less invasive alternative with the potential to reduce surgical risks and offer patients an adjuvant to their treatment, though larger, long-term studies are required to define its role in clinical practice [20].

The rationale for BAE lies in the selective occlusion of the left gastric artery, which supplies the fundus, the primary site of ghrelin production [21]. Reducing blood flow to this region diminishes ghrelin-secreting cell activity and lowers circulating levels of the hormone. This decrease in ghrelin suppresses appetite, promotes earlier satiety, and facilitates weight loss [21]. Unlike surgical resection, BAE preserves gastric anatomy and relies on an endovascular approach using embolic agents such as microspheres to achieve targeted arterial blockade [21]. By modulating hormonal pathways through a minimally invasive technique, BAE offers metabolic benefits comparable to bariatric surgery with potentially fewer complications and shorter recovery times [19].

Anatomy of the left gastric artery

The left gastric artery is the smallest and most superior branch of the celiac trunk, which arises from the abdominal aorta at the T12-L1 vertebral level [22-24]. From its origin, it ascends toward the lesser curvature of the stomach, coursing along its superior aspect. It gives rise to esophageal branches supplying the distal esophagus and gastric branches perfusing the proximal stomach. Along the lesser curvature, it anastomoses with the right gastric artery [22,23].

Although most commonly arising from the celiac trunk, the left gastric artery demonstrates frequent anatomic variation. Reported variants include origins directly from the abdominal aorta or from a common trunk with the inferior phrenic or accessory hepatic arteries [21,22]. Awareness of these patterns is essential during surgical and interventional procedures involving the upper gastrointestinal tract.

The esophageal branches contribute to vascularization of the abdominal esophagus and lower esophageal sphincter, while the gastric branches supply both the anterior and posterior walls of the proximal stomach [22,23,25]. Together, these branches form an extensive vascular network supporting the upper gastrointestinal tract.

Procedure technique

BAE is a minimally invasive procedure aimed at reducing ghrelin-mediated appetite by selectively decreasing blood flow to the gastric fundus. The procedure starts with detailed pre-procedural imaging, including celiac angiography and cone-beam computed tomography (CBCT), to define the vascular anatomy and guide catheter navigation [26,27]. These images help identify the celiac trunk, left gastric artery, and collateral branches, providing a roadmap for selective embolization while minimizing the risk of non-target embolization.

Vascular access is typically obtained through the radial or femoral artery using a 5-F Ultimate Radial or 5-F Jacky catheter [26]. A guidewire is advanced into the celiac trunk, and selective catheterization of the left gastric artery is performed using a microcatheter. In some cases, a second guidewire is used to negotiate tortuous or challenging anatomy. Once the microcatheter is positioned in the mid-left gastric artery, the guidewire is removed to allow precise delivery of embolic material [27].

Embolization is carried out using 300-500 μm compressible Embosphere microspheres, which are injected slowly in repeated aliquots under continuous fluoroscopic guidance [27]. After each injection, contrast flow is monitored over several cardiac cycles to evaluate perfusion to the gastric fundus and confirm that embolization remains confined to the target artery. Serial arterial-phase CBCT is performed throughout the procedure to verify uniform microsphere distribution and an effective reduction in fundal blood flow [26]. This incremental approach helps prevent reflux into adjacent vessels, such as the gastroepiploic or splenic arteries, reducing the risk of unintended ischemia.

Once embolization is complete, the microcatheter is withdrawn, and manual compression is applied to achieve hemostasis at the access site [26,27]. Post-procedural care usually involves overnight observation, with an initial gastroscopy performed before discharge to assess the gastric mucosa. A second gastroscopy is typically scheduled one week later to evaluate fundal ischemia and confirm the effectiveness of the procedure [26-29]. This systematic approach provides a safe and reproducible method for BAE and allows for consistent reporting of outcomes.

Clinical outcomes and evidence review

BAE has emerged as a minimally invasive, hormonally targeted intervention for obesity, producing modest yet clinically meaningful weight loss while maintaining a favorable safety profile compared with traditional bariatric surgery. Early studies confirmed the procedure’s feasibility and technical safety. The BEAT Obesity trial reported a mean excess weight loss of 8-12% at 12 months post-embolization, with no major procedure-related complications [28], and subsequent cohort studies demonstrated sustained weight reduction along with improvements in metabolic parameters, including glycemic control and lipid profiles [29,30]. 

BAE appears particularly beneficial for patients ineligible for conventional bariatric surgery or preferring less invasive approaches. Evidence suggests that weight loss magnitude correlates with the extent of fundal embolization, although standardized procedural protocols remain under investigation [31]. Adverse events are generally mild and transient, including abdominal discomfort, nausea, or minor gastric ulceration, with serious complications being rare [28,29]. 

Short-term outcomes are encouraging. Weiss et al. (2017) reported that five patients achieved a mean excess weight loss of 5.9% at one month and 9.0% at three months, accompanied by significant reductions in serum ghrelin levels, supporting appetite suppression as a primary mechanism [28]. Longer-term data are limited but promising. Pirlet et al. (2020) observed a median weight loss of 11 kg (7.7% of body weight) sustained over two years, with only minor, self-limited complications; one patient required repeat embolization due to spontaneous recanalization [8]. 

Overall, BAE represents a safe and effective adjunct or alternative to conventional weight-loss strategies. Its minimally invasive, hormonally targeted approach shows potential for both short- and long-term obesity management. Continued investigation is warranted to refine procedural protocols, evaluate the durability of effect, and compare outcomes with established surgical interventions [32].

Comparison with bariatric surgery

BAE and traditional bariatric surgeries, such as RYGB and SG, differ in weight-loss efficacy, metabolic outcomes, safety profiles, and patient eligibility. RYGB typically yields the most significant weight loss, with patients achieving 60-80% excess weight loss (EWL) within 12-24 months. A meta-analysis demonstrated that RYGB provides superior weight reduction compared with SG [31,32]. SG also results in substantial weight loss, with 50-70% EWL over a similar period; however, it has been associated with higher rates of gastroesophageal reflux and marginally increased complication risks compared with RYGB [30]. In contrast, BAE offers more modest reductions of approximately 5-12% EWL at 12 months, but as a minimally invasive procedure, it avoids the risks of general anesthesia and extensive surgery [32].

Metabolic improvements follow a similar pattern. RYGB is highly effective for type 2 diabetes remission and improvement of hypertension and hyperlipidemia [28,32]. SG also improves these parameters, though to a slightly lesser extent [33,34]. BAE has shown moderate benefits in glycemic control and lipid profiles, supporting its potential as a less invasive option for metabolic management [33].

Safety profiles further distinguish these interventions. Surgical procedures carry risks such as leaks, bleeding, nutritional deficiencies, and long-term complications [34]. BAE, by contrast, is generally well-tolerated, with mostly mild and transient side effects such as abdominal discomfort or nausea [35]. This favorable safety profile makes BAE suitable for patients who are not surgical candidates or prefer non-operative approaches [31,32].

Patient selection and procedural considerations are important. RYGB and SG are recommended for patients with BMI ≥40 kg/m² or ≥35 kg/m² with comorbidities [34], while BAE may be appropriate for those with BMI 25-40 kg/m² who seek a less invasive option [35]. Recovery is faster with BAE, though long-term weight-loss durability remains under investigation [34,35]. 

In summary, as depicted in Table 2, RYGB and SG provide more substantial and sustained weight loss with pronounced metabolic benefits, but BAE offers a safe, minimally invasive alternative for selected patients. Each approach has distinct advantages and limitations, and the choice should be tailored to the patient's needs, preferences, and overall health status. Continued research will clarify the long-term efficacy and optimal indications for BAE relative to surgical options [33,35].

**Table 2:** Comparative Summary of Bariatric Arterial Embolization and Traditional Bariatric Surgeries. Summary of key features of bariatric arterial embolization (BAE), Roux-en-Y gastric bypass (RYGB), and sleeve gastrectomy (SG), including weight-loss efficacy, metabolic benefits, safety, invasiveness, patient selection, recovery, and durability of effect. BAE is minimally invasive with modest weight loss and a favorable safety profile, while RYGB and SG achieve greater and more sustained weight reduction but carry higher procedural risks [34,35].

Feature	Bariatric Arterial Embolization (BAE)	Roux-en-Y Gastric Bypass (RYGB)	Sleeve Gastrectomy (SG)
Weight Loss (EWL)	5–12% at 12 months [34,35]	60–80% within 12–24 months [34]	50–70% within 12–24 months [34]
Metabolic Benefits	Moderate improvements in glycemic control and lipid profile [35]	High rate of T2DM remission, improved hypertension and lipids [32,34]	Moderate-high metabolic improvement; slightly lower than RYGB [34]
Safety Profile	Generally mild, transient side effects (abdominal pain, nausea) [32,35]; serious complications rare	Risk of leaks, bleeding, nutritional deficiencies, long-term complications [34]	Risk of leaks, staple-line bleeding, GERD exacerbation [34]
Invasiveness	Minimally invasive; no general anesthesia required [35]	Major surgery; requires general anesthesia [34]	Major surgery; requires general anesthesia [33,34]
Patient Selection	BMI 25–40 kg/m², unsuitable for surgery, or seeking a less invasive approach [35]	BMI ≥40 kg/m² or ≥35 kg/m² with comorbidities [34]	BMI ≥40 kg/m² or ≥35 kg/m² with comorbidities [34]
Recovery Time	Short, outpatient or 1–2 day observation [35]	Longer, typically weeks to months [34]	Longer, typically weeks to months [34]
Durability of Weight Loss	Limited long-term data; promising [34]	Sustained long-term weight loss [34]	Sustained long-term weight loss, slightly less than RYGB [34]

Future directions and research gaps

Given early evidence of feasible weight loss, BAE is a promising adjunct to lifestyle therapy and a potential intermediate option between pharmacotherapy/endoscopic therapies and surgical bariatric procedures. However, BAE remains significantly understudied; robust investment is needed to define long-term efficacy and safety. Current studies are limited by small sample sizes, resulting in low statistical power and wide confidence intervals [19]. Additional limitations include loss to follow-up, variable adherence to pre- and post-procedural adjunctive therapies, and demographic imbalances with racial over-representation [19].

When compared to GLP-1 receptor agonists, BAE offers a potentially durable, procedure-based alternative that does not rely on chronic medication use or the ongoing costs and compliance challenges associated with pharmacotherapy [14,16]. However, GLP-1 agents currently have a more robust evidence base and are reversible, whereas the long-term metabolic effects of BAE remain to be clarified [14]. In contrast, endoscopic sleeve gastroplasty achieves greater short-term weight loss than reported in early BAE trials; however, it is more invasive and requires anesthesia and endoscopic suturing expertise [17]. BAE’s appeal lies in its minimally invasive, hormonally targeted approach, which may complement these modalities rather than compete with them, especially for patients who are poor surgical candidates or unable to tolerate long-term pharmacologic therapy [16-19].

These shortcomings contribute to imprecise effect estimates, under-detection of uncommon harms, and limited generalizability [17,19]. Future research should prioritize adequately powered, sham-controlled randomized trials; longer follow-up (beyond 12 months); and standardized procedural techniques and outcome measures [17,19]. Comparative-effectiveness studies against current anti-obesity pharmacotherapy and endoscopic options are also warranted. It would also be significant to research the implementation and cost-effectiveness of BAE, particularly given the disproportionate burden of obesity in lower socioeconomic groups [17]. Such methods will isolate the causal effect of BAE and yield reliable, generalizable estimates to guide clinical adoption.

## Conclusions

BAE represents a promising addition to the spectrum of obesity management strategies. By harnessing minimally invasive, hormonally targeted mechanisms, BAE offers a novel approach that bridges the gap between conservative interventions and traditional bariatric surgery. Its appeal lies in the ability to modulate appetite and support weight loss while avoiding many of the risks and recovery demands associated with surgical procedures. Although current evidence points to modest but meaningful short-term benefits, the long-term durability of outcomes remains to be fully determined. Importantly, BAE may expand treatment options for patients who are ineligible for surgery, prefer less invasive therapies, or wish to combine procedural interventions with lifestyle modifications. As research continues to clarify optimal techniques, patient selection criteria, and long-term efficacy, BAE has the potential to play a significant role in personalized, patient-centered approaches to obesity care.
